# Knowledge on Irradiation, Medical Imaging Prescriptions, and Clinical Imaging Referral Guidelines among Physicians in a Sub-Saharan African Country (Cameroon)

**DOI:** 10.1155/2017/1245236

**Published:** 2017-05-23

**Authors:** Boniface Moifo, Ulrich Tene, Jean Roger Moulion Tapouh, Odette Samba Ngano, Justine Tchemtchoua Youta, Augustin Simo, Joseph Gonsu Fotsin

**Affiliations:** ^1^Department of Radiology and Radiation Oncology, Faculty of Medicine and Biomedical Sciences, The University of Yaoundé I, Yaoundé, Cameroon; ^2^Radiology Department, Yaoundé Gynaecology, Obstetrics and Pediatrics Hospital (YGOPH), P.O. Box 4362, Yaoundé, Cameroon; ^3^Radiology Department, Yaoundé University Teaching Hospital, Yaoundé, Cameroon; ^4^Department of Radiation Oncology, Yaoundé General Hospital, Yaoundé, Cameroon; ^5^National Radiation Protection Agency (NRPA), Yaoundé, Cameroon

## Abstract

**Background:**

Clinical imaging guidelines (CIGs) are suitable tools to enhance justification of imaging procedures.

**Objective:**

To assess physicians' knowledge on irradiation, their self-perception of imaging prescriptions, and the use of CIGs.

**Materials and Methods:**

A questionnaire of 21 items was self-administered between July and August 2016 to 155 referring physicians working in seven university-affiliated hospitals in Yaoundé and Douala (Cameroon). This pretested questionnaire based on imaging referral practices, the use and the need of CIGs, knowledge on radiation doses of 11 specific radiologic procedures, and knowledge of injurious effects of radiation was completed in the presence of the investigator. Scores were allocated for each question.

**Results:**

155 questionnaires were completed out of 180 administered (86.1%). Participants were 90 (58%) females, 63 (40.64%) specialists, 53 (34.20%) residents/interns, and 39 (25.16%) general practitioners. The average professional experience was 7.4 years (1–25 years). The mean knowledge score was 11.5/59 with no influence of sex, years of experience, and professional category. CIGs users' score was better than nonusers (means 14.2 versus 10.6; *p* < 0.01). 80% of physicians (124/155) underrated radiation doses of routine imaging exams. Seventy-eight (50.3%) participants have knowledge on CIGs and half of them made use of them. “Impact on diagnosis” was the highest justification criteria follow by “impact on treatment decision.” Unjustified requests were mainly for “patient expectation or will” or for “research motivations.” 96% of interviewees believed that making available national CIGs will improve justification.

**Conclusion:**

Most physicians did not have appropriate awareness about radiation doses for routine imaging procedures. A small number of physicians have knowledge on CIGs but they believe that making available CIGs will improve justification of imaging procedures. Continuous trainings on radiation protection and implementation of national CIGs are therefore recommended.

## 1. Introduction 

The medical use of ionizing radiation is becoming the most significant man-made source of exposure for the population in the western world and also in developing countries [1, 2]. In a community-based level, to reduce the burden of medical imaging radiation, we need to apply the two cornerstones of radiation protection of patient which are justification and optimization of exposures. This concerns all the steps of the imaging process starting from the elaboration of the request forms by referring physicians to the validation, realization, and interpretation of the imaging examinations. The last two are those that deserve the quality of the imaging procedure and are on the responsibility of radiographers and radiologists. The referring physician has the responsibility to prescribe the best imaging procedure for the patient clinical condition, giving the clinical and administrative data useful for the validation of that choice. Many countries develop or adopt clinical imaging guidelines (CIGs) to support the physicians' prescription of the most suitable imaging procedure for the patient clinical condition [3–5]. These CIGs are justification based tools expressing for a given clinical setting possible imaging procedures and for each imaging procedure the level of indication, the level of scientific proof (reliability and accuracy), the level of irradiation, and some comments (procedure for substitution, etc.). Hence, referring clinicians must be aware of the level of patients' doses and the possible harmful effects of this exposure in order to justify irradiating medical imaging procedure.

In Cameroon, there are evidences of the lack of completeness of imaging request forms [6, 7]. Many studies on radiation protection established the poor knowledge of medical professionals on standards and principles of radiation protection [6, 8, 9]. This is enhanced by the lack of training on radiation protection and the absence of “guide for better use of medical imaging procedure” [10]. Various studies from different parts of the world have demonstrated general lack of knowledge on radiation doses and their adverse potential in the absence of referral guidelines among referring physicians [2, 11–19]. This situation in many developing countries is unlikely to be as much as this [11, 20]. The level of awareness concerning radiation doses of a given imaging exam influences clinician behaviors [21]. If they do not have enough information related to radiation doses and safety, their actions will not be safe and will result in adverse effects.

As part of a project to improve best clinical practices, with a view of clinicians' awareness on radiation risk and justification of the request of medical imaging procedures, we conducted a survey to investigate the radiation dose knowledge and use of CIGs among physicians in relation to their referral practice in a pool of seven first reference hospitals in Cameroon.

## 2. Materials and Methods

This cross-sectional study was conducted in seven university-affiliated hospitals in Yaoundé and Douala in Cameroon from July to August 2016. Confidentiality and anonymity were maintained according to the regulations mandated by Research Ethics Committee of Faculty of Medicine and Biomedical Sciences of University of Yaoundé I, in accordance with the Declaration of Helsinki.

### 2.1. Target

Recruitment was done by convenience sampling of all physicians who were consulting during this period on a voluntary basis. The participants were informed that the results would be used only for a scientific study.

### 2.2. Questionnaire

A self-administered questionnaire was filled by the physician in the presence of the investigator who collected it immediately. This questionnaire was based on a model used by Borgen and Stranden in Norway in 2014 [12]. The questions concerned reasons for referring for imaging procedures, knowledge and use of clinical imaging referral guidelines (CIGs), knowledge on radiation doses of 11 most prescribed medical imaging procedures, knowledge on stochastic and deterministic effects of radiation, the need of training on ionizing radiation, and the need of CIGs. We removed questions for which the answers were not yet available in our milieu like those on the source of radiation or had other items for the better understanding of national specificities such as past training on justification or radioprotection and the facilities support of CIGs. Age, gender, professional category, place of degree graduation, and years of professional experience of the respondents were also registered.

### 2.3. Statistical Analysis

We constructed a total radiation knowledge score ranging from 0 to 59 following the quotation used by Borgen and Stranden in Norway [12]. For the imaging procedures, a correct answer was coded 3 points, each yielding a maximum of 33 points. Knowing the existence of adverse effects of radiation was coded 2 points; the value of a radiation dose of two chest X-ray incidences and international unit of the effective radiation dose were coded 4 points each. Knowing the terms stochastic and deterministic was coded 4 points, and correct categorization of the six detrimental effects correctly was coded 2 points each, resulting in a maximum of 16 points. Missing data gave 0 points, and a total radiation knowledge score was noted for all participants.

Data were analyzed using the Statistical Package for the Social Sciences for windows (SPSS 19.0) to determine descriptive statistics including frequency distribution, mean, standard deviation, and percentages. Level of knowledge, attitude, and practices were calculated as a percentage of correct answers in each section. Levels less than 50% were considered poor knowledge. For comparison, we used ANOVA test or Student's *t*-test and Chi square test of Pearson or Fischer for means and frequencies, respectively, between professional category, level of experience, and services when it was suitable.

## 3. Results

### 3.1. Demographics

Of the 180 participants solicited, 155 questionnaires (86.1%) were completed. Twenty-five participants did not consent to participate to the survey. The mean age (range) of participants was 34.8 (23–56) years with female predominance (58%). The average experience of all physicians including postgraduation years was 7.4 years (1–25 years). The main hospital unit of interviewed physicians was Internal Medicine (68/155). Sixty-three respondents (40.7%) were specialists.  Table 1 shows the distribution of general characteristics of respondents according to their professional category.

### 3.2. Global Knowledge Score of Respondents on Irradiation

The mean total knowledge score was 11.5/59 (11.3 for men and 11.7 for women). Gathering respondents into groups based on clinical experience of more than or less than 10 years, the means scores were 11.6 and 11.0, respectively.  Table 2 shows the mean global knowledge score of physicians with regard to sex, years of experience, site of training, and their working units. Differences between these groups were not statistically significant.

### 3.3. Knowledge on Irradiation during Some Imaging Procedures

All the respondents recognized that ionizing radiation has dissimilar injurious effects on patients; 95.5% of them had mistaken the effective radiation international dose unit (millisievert). None of them were able to give the approximate dose of a frontal chest X-ray exam (≈0.05 mSv) which is the most prescribed X-ray procedure.

More than 85% of the physicians undervalued radiation doses (in terms of number of chest X-ray doses) of routine imaging procedures: 85.8 to 86.5% underrated the doses of lumbar, abdominal, and thoracic CT scans, while 88.4 to 98.7% underrated the doses of intravenous pyelography, barium enema, lumbar spine, and pelvic X-rays. Magnetic resonance imaging (MRI) and ultrasound were considered as radiating procedures, respectively, by 83% and 28% of respondents.

Only 25 (16.1%) out of 106 clinicians knowing that X-rays had harmful effects declared that they were familiar with deterministic and stochastic terms. When sorting six harmful effects of X-rays as either stochastic or deterministic, these 25 clinicians' mean score (correct answers) was 48% (Table 3).

### 3.4. Physicians' Perception on Their Prescriptions of Medical Imaging Procedures and the Use and Need of Clinical Referral Imaging Guidelines

Regarding the use of clinical referral guidelines for imaging, we found that 78 (50.3%) respondents have knowledge on referral guidelines and only a half of them (36) had made use of it. Overall, there was no statistically significant difference concerning the use of guidelines (*p* > 0.05) among clinicians in different categories, experience, place of training, and hospital units of work. 72.9% of participants reported that they never had training on radiation protection since their graduation. Seventy-six physicians (49%) defined clinical imaging referral guidelines (CIGs) as a tool for limitation of dose, while 23.2% defined them as a tool for justification.

**Figure 1:**
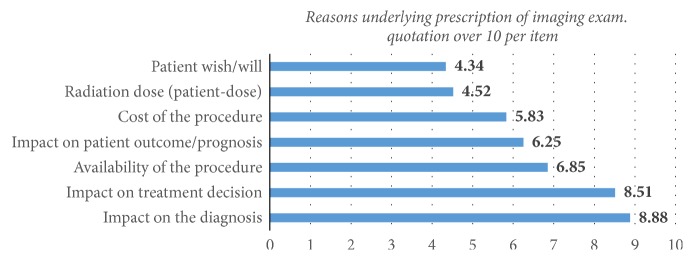
Weighting of physicians' reasons for the prescription of imaging procedure.

Among the reasons underlying the request of an imaging exam (Figure 1), “impact on the diagnosis” was the highest weighted, with a mean score of 8.8/10 (median: 9). The importance of “patient's wish” was the lowest (mean: 4.3/10; median: 4), followed by considerations on “radiation dose to patient” (mean: 4.52/10; median: 4). The choice of a given imaging technique when prescribing was sustained by the basic knowledge acquired during medical training for 60% or by their personal experience for 20% of respondents.

**Figure 2:**
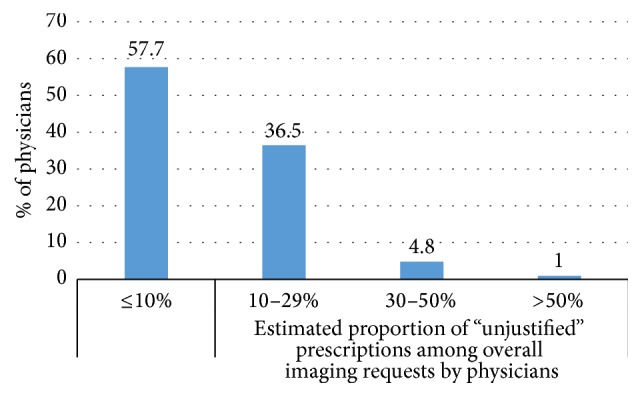
Proportion of imaging prescriptions considered by physicians unlikely to affect treatment/diagnosis.

**Figure 3:**
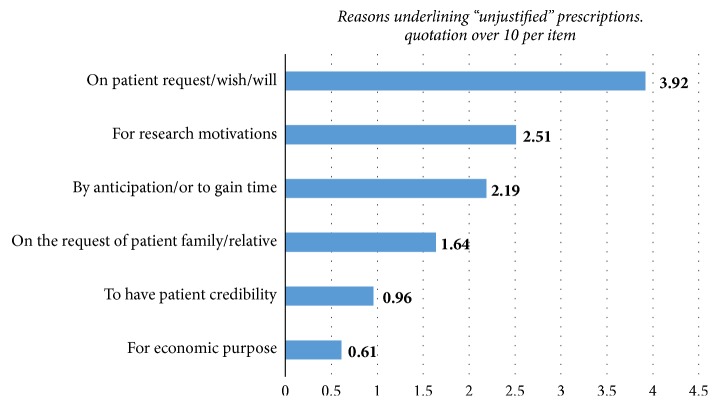
Physicians' reasons for prescriptions of imaging procedures unlikely to affect treatment/diagnosis.

Despite the fact that “impact on diagnosis or treatment” was the main reason for the request of imaging procedures, nearly two-thirds (67.1%) of physicians declared that they sometime requested for imaging that would not likely have an effect on treatment/diagnosis. The proportion of these requests represented less than 10% of overall prescriptions for 57.7% of the physicians (Figure 2). The main reason for these prescriptions was the “patient expectation or will” weighted 3.9 on 8 (median: 4) followed by “research motivations” with 2.5 (median: 2) as shown in Figure 3.

Concerning physicians' self-perception on the need of training on radiation protection, 71% of respondents agreed to have had a training on justification and 27% on radiation protection. Nearly two-thirds (67.7%) of physicians claimed to have been sensitized on patient irradiation in diagnostic imaging. There is a wish for 95.5% of physicians to dispose national clinical imaging guidelines. All participants thought that making available CIGs will improve their knowledge and then their behavior regarding prescriptions of imaging procedures. The most suitable CIGs support preferred by our respondents was a didactic version for computer, smartphone, or tablet (32.2%) followed by smart software version (23.2%).

### 3.5. Knowledge on Irradiation in Relation with Imaging Requests and Use of CIGs

Total knowledge score on irradiation was better in respondents using CIGs than those who did not use CIGs (average score: 14.2 versus 10.6, *p* < 0.01). However, this knowledge score was similar: (1) among respondents who had training on justification (*n* = 110) compared to those who did not have training (*n* = 45) (average score: 11.6 versus 11.1, *p* = 0.67) and (2) among respondents who had training in radiation protection (*n* = 42) compared to those who did not have training (*n* = 113) (average score: 12.7 versus 11.0, *p* = 0.15) and (3) for physicians who admitted (*n* = 100) to sometimes prescribe imaging tests unlikely to affect treatment/diagnosis compared to those who denied (*n* = 55) (average score: 11.7 versus 10.9, *p* = 0.46).

## 4. Discussion

### 4.1. Strength and Limitations

This is a first ever study from Cameroon among referring clinicians on their attitude and knowledge concerning radiation exposure of patients during common radiology investigations and their familiarity to the irradiation doses and exposure dose unit and self-assessment of their medical imaging requests. However, the use of questionnaires has inherent limitations, as some answers reflect respondents' subjective opinions. In the present study, respondents' self-report on their own practice should be interpreted with care. The immediate filling of the questionnaire in the presence of the investigator avoided responses after consultation of documents. The anonymity of the questionnaire raised complexity related to judgment of an individual respondent. The quotation points of open questions and those related to the practice allowed having less subjective responses and the possibility of appraising practical attitudes. Nevertheless, national generalization of results of this study should be raised carefully due to the impossibility of randomly choosing clinicians. But up to 86% respondents seen in their office is a nonnegligible participation rate. The predominance of specialists reflects the normal distribution of the medical professional population at the university-affiliated hospitals in the country.

### 4.2. Global Radiation Knowledge Score of Respondents

Our study had shown that, in Cameroon, clinicians had poor knowledge on radiation with a worse mean score less than 20% (11.5/59) [12, 14–20, 22–28]. All the three professional respondent groups possessed the same very low knowledge on radiation. Even though in agreement with previous reports, it was noteworthy that only 23% of the clinicians had used clinical imaging referral guidelines (CIGs), which is in the range of the level of use in other studies (10–40%) [20, 29]. Two main reasons of this, as worldwide demonstrated, are the absence of diffused national CIGs and the insufficiency of training on justification as it was reported in other studies in the same settings [7, 10]. This could corroborate some findings of our study. Firstly, the general knowledge was worse in clinicians who said they did not use referral guidelines. Secondly, neither the self-reported participations to specific training on justification (71% of clinicians) nor those on radiation protection (27%) significantly modified their knowledge on irradiation. Lastly, the poor general knowledge of doctors seems not to be link to the high rate of imaging prescriptions unlikely to have an effect on therapeutic decision.

### 4.3. Specific Knowledge on Irradiation during Imaging Procedures

Like in other countries in Africa and in the world, this study confirmed the underestimation of irradiation dose of the most prescribed imaging exams by doctors in Cameroon [12, 20, 23, 30–32]. Despite the fact that they had poor knowledge on irradiation, all of them recognized the dissimilar injurious effect of ionizing radiation of some imaging modalities. This reveals their superficial knowledge on the topic, probably acquired during medical training.

In fact, critical findings obtained during this study sustained this superficial knowledge. For all the imaging procedures evaluated in this study, none of the participants were able to have 50% of the correct range of patient-dose for common imaging procedures. The proportion (for all procedures) of respondents with false answers in this study was the highest one ever obtained in a developing country to the best of our knowledge, with a global tendency of all participants (like elsewhere) to the underestimation [12, 20, 23, 29, 30]. Surprisingly, we found a high proportion (up to 80%) of clinicians thinking that MRI is an ionizing exam [12, 13, 31]. Some authors like Moifo et al. attributed this to the new integration of MRI in our country [10]. But this high proportion compared to other studies conducted in developing countries suggests that things are becoming worse, maybe due to the rarity of this modality (just three medical centers having MRI machine for about 25 million inhabitants). This statement cannot be transposed to ultrasound procedures which are widespread in Cameroon but 28% of respondents considered it as an ionizing technic with a radiation dose up to 1–10-folds higher than one chest X-ay (modal class). We think that this could be due to very superficial knowledge on radiation issue doubled by the insufficiency of training on radiation protection. For instance, they could not be able to estimate the radiation dose of a complex imaging technic if they could not state correctly the international radiation unit (95.5% of wrong answers) or if they were not able to state properly (no good answer) the radiation dose of a single chest X-ray.

Concerning adverse effect of imaging radiation, physicians are not comfortable with the terms stochastic and deterministic, and only 48% were able to dissociate stochastic from deterministic harmful effects of radiation. And even this could be due to the 50% probability to randomly choose between deterministic or stochastic on this question. It could be different if we had formulated the question a little bit different with an open answer. Nevertheless, this confirms the approximate knowledge of the respondents.

### 4.4. Self-Perception of Imaging Prescriptions and Use and Need of Referral Guidelines

The imaging referral practices of respondents reflect clearly their level of awareness concerning clinical imaging issue [21]. For instance, the poor level of knowledge concerning patient-dose during imaging procedure is confirmed by the poor consideration to “radiation dose” as criteria influencing the choice of a given imaging procedure. The level of irradiation should contribute to the justification of an imaging procedure alongside with the impact on diagnosis and the therapeutic decision [3–5]. Furthermore, this study reveals a very high proportion of clinicians erquesting for imaging tests whose results are unlikely to affect patients' treatment/diagnosis, like in some developing countries [20, 31] but in greater proportion than in developed countries [12, 29]. But in our settings, respondents argue their choice by facing the burden of patients environment (family, friend, or other relatives), helping them to grant some time for elaborating diagnostic or for building some notion by making research. All these reasons could be avoided if they have in disposition a tool helping them like CIGs for better prescription of imaging examinations. Instead, we found that only half of the physicians knew the existence of CIGs and less than one-quarter had ever used it. Similar proportions have been described in developed and developing countries [20, 29]. It had been shown that incorporating the imaging guideline tools in the process of justification of imaging referral reduces significantly the proportion of useless examinations in imaging diagnostics [2]. When we ask for the type of facility tools the respondents may like to possess in light of making it easier to use the guidelines, the choice focused on didactic version for computer, smartphone, or tablet followed by smart software version. This means that the better way to spread CIGs in our environment should integrate ITC (information technology and communication).

## 5. Conclusion

The level of awareness of clinicians in Cameroon of irradiation during imaging procedure is very low and superficial and this affects the quality of their imaging prescriptions. This is enhanced by the lack of training on radiation protection and clinical imaging referral guidelines (CIGs). Improving justification of imaging request may pass through recurrent training on radiation protection to both undergraduates and postgraduates clinicians and by adopting national CIGs, followed by the vulgarization of these national CIGs through ITC.

## Figures and Tables

**Table 1:** General characteristics of respondents according to their professional category.

General characteristics	Interne/residents *n* = 53	General practitioners *n* = 39	Specialists *n* = 63	*p* value
Mean age in years (SD^*∗*^)	30.6 (3.78)	31.6 (4.8)	40.2 (6.3)	**<0.01 **
Sex, *n* = M/F (%)	17/36 (**40.7**)	12/27 (34.2)	36/27 (25.1)	**<0.01**
Mean years of experience (SD^*∗*^)	4.2 (2.7)	4.4 (3.6)	**7.4** (5.8)	**<0.01**
Place of training degree: national/abroad (% of abroad)	46/7 (13.2)	29/10 (25.6)	44/19 (30.2)	**0.09**
*Hospital working units of respondents, n (% in the professional category)*				
Internal medicine, *n* = 68	24 (45.3)	17 (43.5)	27 (42.9)	**<0.01**
Surgery, *n* = 32	9 (17)	4 (10.3)	19 (30.2)	
Gynecology, *n* = 22	9 (17)	4 (10.3)	9 (14.3)	
Pediatric, *n* = 20	10 (18.9)	3 (7.7)	7 (11.1)	
Emergency, *n* = 13	1 (1.9)	11 (28.2)	1 (0.6)	

^*∗*^Standard deviation.

**Table 2:** Global knowledge of physicians with regard to their general characteristics.

General characteristics	Global knowledge score		*p* value
	Mean	SD^*∗*^	
Sex: male/female	11.7/11.3	7.0/6.7	0.68
Place of degree training: national/abroad	12.0/11.0	6.9/6.5	0.08
Years of experience: <10 years/≥10 years	11.6/11.0	7.0/6.5	0.66

Resident or interne physician	12.0	7.5	0.18
Specialist physician	11.5	6.9	
General practitioners	10.3	5.6	

Physician in emergency unit	13.0	9.5	0.84
Physician in internal medicine unit	11.7	7.3	
Physician in surgery unit	11.1	6.1	
Physician in gynecology unit	11.2	4.8	
Physician in pediatric unit	10.3	6.8	

^*∗*^Standard deviation.

**Table 3:** Respondents (*n* = 25) matching responses with deterministic and stochastic harmful effects of X-rays.

Type of effects	Stochastics, *n* (%)	Deterministic, *n* (%)
Infertility	9 (36)	16 (64)
Leukemia	12 (48)	13 (52)
Genetic abnormality	13 (52)	12 (48)
Fetal malformation	14 (56)	11 (44)
Radiation-induced cancer	16 (64)	9 (36)
